# Effects of Protease, Phytase and a *Bacillus sp*. Direct-Fed Microbial on Nutrient and Energy Digestibility, Ileal Brush Border Digestive Enzyme Activity and Cecal Short-Chain Fatty Acid Concentration in Broiler Chickens

**DOI:** 10.1371/journal.pone.0101888

**Published:** 2014-07-11

**Authors:** Ganapathi R. Murugesan, Luis F. Romero, Michael E. Persia

**Affiliations:** 1 Department of Animal Science, Iowa State University, Ames, Iowa, United States of America; 2 Danisco Animal Nutrition - DuPont Industrial Biosciences, Marlborough, United Kingdom; National Institute of Agronomic Research, France

## Abstract

Two experiments were conducted to determine the effects of protease and phytase (PP) and a *Bacillus sp*. direct-fed microbial (DFM) on dietary energy and nutrient utilization in broiler chickens. In the first experiment, Ross 308 broiler chicks were fed diets supplemented with PP and DFM in a 2×2 factorial arrangement. The 4 diets (control (CON), CON + PP, CON + DFM, and CON + PP + DFM) were fed from 15–21 days of age. In Experiment 1, significant interaction (*P*≤0.01) between PP and DFM on the apparent ileal digestibility coefficient for starch, crude protein, and amino acid indicated that both additives increased the digestibility. Both additives increased the nitrogen retention coefficient with a significant interaction (*P*≤0.01). Although no interaction was observed, significant main effects (*P*≤0.01) for nitrogen-corrected apparent ME (AMEn) for PP or DFM indicated an additive response. In a follow-up experiment, Ross 308 broiler chicks were fed the same experimental diets from 1–21 days of age. Activities of ileal brush border maltase, sucrase, and L-alanine aminopeptidase were increased (*P*≤0.01) by PP addition, while a trend (*P* = 0.07) for increased sucrase activity was observed in chickens fed DFM, in Experiment 2. The proportion of cecal butyrate was increased (*P*≤0.01) by DFM addition. Increased nutrient utilization and nitrogen retention appear to involve separate but complementary mechanisms for PP and DFM, however AMEn responses appear to have separate and additive mechanisms.

## Introduction

Supplementation of broiler diets with exogenous enzymes results in increased dietary energy and protein utilization through increased substrate availability [1]. The amount of anti-nutritive factors such as non-starch polysaccharides, protease inhibitors, lectins and phytate present in a corn-soybean meal (SBM)-dried distillers grains with solubles (DDGS)-based diet presents an ideal opportunity to use exogenous enzymes. Although a vast amount of literature is available on the effects of carbohydrases on dietary energy release and their interaction with phytase [2], data on the effects of protease and protease in combination with phytase are scarce. Inclusion of protease alone in broiler diets has to date produced mixed responses [3], [4], although consistently increased performance due to phytase addition has been reported [5]. Ghazi et al. [4] observed increased nitrogen (N) retention in broiler chickens fed protease treated SBM. Protease supplementation has been employed to lower dietary protein level without a reduction in broiler performance [6]. Supplemental phytase has been observed to increase the activity of pepsin, H^+^K^+^ATPase, trypsin and aminopeptidase [7]. Ravindran et al. [8] reported consistently increased N retention in broiler chickens fed supplemental phytase, independent of dietary phytic acid and non-phytate phosphorus levels. Hence the effects of exogenous protease and phytase combination (PP) in broiler diets on dietary nutrient utilization needs to be assessed.

Secondly, *Bacillus sp*. have been one of the most widely researched direct-fed microbial (DFM) as an alternative to antibiotic growth promoters in poultry diets. Specific *Bacillus sp*. strains have been observed to change the gastro-intestinal microbial profile and contribute to the reduction of pathogens, resulting in increased broiler performance [9]. However, the effect of *Bacillus sp*. in increasing dietary nutrient utilization efficiency of birds is of focus lately owing to higher feed costs. Santoso et al. [10] reported increased nutrient digestion and utilization in broiler chicks due to supplementation with a *Bacillus* DFM, which the authors speculated to the secretion of protease, amylase and lipase by the DFM. Supplementation of *Bacillus sp*., has been reported to increase total short-chain fatty acids (SCFA) and acetate concentration in the ceca of broiler chickens [11]. In 42 day old broiler chickens, *Bacillus* DFM lowered the deposition of abdominal fat while increasing body weight and feed efficiency indicating efficient nutrient utilization [12]. Hooge et al. [13] reported increased body weight and reduced feed conversion ratio for chicks fed *Bacillus* DFM in a series of experiments. Although PP or *Bacillus* DFM have been demonstrated independently to increase the efficiency of nutrient utilization, it is not clear the extent to which direct digestion of substrates, changes on microbial communities, or immune effects are responsible for effects on performance and nutrient utilization.

Two experiments were conducted to determine the effects of PP and DFM on the apparent ileal digestibility coefficient (AIDC) of starch, crude protein (CP) and amino acids (AA), N retention and N corrected apparent ME (AMEn) in Experiment 1. The follow-up experiment explored the possible mechanisms through which PP and DFM affect the nutrient digestibility and utilization, including ileal brush border activity of digestive enzymes - maltase, sucrase and L-alanine aminopeptidase (LAAP), and concentration of cecal SCFA such as acetate, propionate and butyrate. The hypothesis evaluated was that supplementation of PP and DFM to broiler chickens would increase the dietary energy and nutrient digestibility by independent modes of action resulting in additive responses.

## Materials and Methods

Two experiments were conducted to evaluate the effects of PP and DFM on broiler chickens, at the Poultry Research and Teaching Unit of Iowa State University. All animal procedures were approved by the Institutional Animal Care and Use Committee of Iowa State University before the start of the experiments.

### Animals and housing

In the first experiment, Ross 308 male broiler chicks (Aviagen Inc., AL) were fed a standard starter diet, formulated to meet or exceed the breeder recommendations, from 1–14 days and experimental diets from 15–21 days. On day 15, chicks were individually weighed, sorted by weight, wing banded, and allocated to cages based on body weight to achieve uniform initial weight among cages and treatments. This resulted in 8 batteries (experimental units) of 6 chicks (568 cm^2^/chick) for each of the 4 treatments. Experimental diets were randomly assigned to batteries in a completely randomized design. Chicks were located within an environmentally controlled room, received supplemental heat starting at 35°C and decreasing by 2°C weekly. The photoperiod provided was 23 hr of light and 1 hr of darkness. Chicks were provided *ad libitum* access to feed and water, and monitored twice daily throughout the experimental period. Mortalities were removed, weighed, and recorded as they occurred.

In the second experiment, day-old, Ross 308 male broiler chicks (Aviagen Inc., AL) were individually weighed, sorted by weight, wing banded and allocated to floor pens based on body weight to achieve uniform initial weight among the treatments. Each of the 4 dietary treatments were comprised of 10 chicks per pen. Each chick in the same pen was considered as an experimental unit. Chicks were fed experimental diets from 1–21 days. All other management practices were similar to Experiment 1 with the exception that chicks were housed in floor pens (0.15 m^2^/chick).

### Diets

Both experiments utilized the same diets organized in a 2×2 factorial arrangement both with and without PP and DFM supplementation. This factorial resulted in the following 4 treatments, 1. Control (CON); 2. CON + PP; 3. CON + DFM; and 4. CON + PP + DFM). The un-supplemented CON diet was corn-SBM-DDGS-based, and formulated to meet or exceed the breeder recommendations [14], except for a 0.94 MJ/kg reduction in ME (Table 1). Since the same experimental diets were fed from 1–21 days in the second experiment, the dietary ME reduction was 0.42 MJ/kg of diet from 1–10 days, and 0.94 MJ/kg of diet from 11–21 days in comparison to breeder recommendations [14]. The PP used was a combination of a serine protease (EC 3.4.21.62) from *Bacillus subtilis* (5,000 units/kg of diet) and phytase from *Buttiauxela sp*. (550 FTU/kg of diet), while the DFM contained spores (8×10^5^ CFU/g of diet) of a specific *Bacillus licheniformis* strain (Danisco Animal Nutrition - DuPont Industrial Biosciences, Marlborough, UK). One protease unit was defined as the amount of enzyme that releases 1.0 µg of phenolic compound, expressed as tyrosine equivalents, from a casein substrate per minute at pH 7.5 and 40°C. One phytase FTU was defined as the quantity of enzyme that releases 1 µmol of inorganic phosphorus per min from 0.15 mM sodium phytate at pH 5.5 at 37°C. Both PP and DFM were included at a dose of 500 mg/kg of diet as recommended by the manufacturer. Titanium dioxide (TiO_2_) was added at the rate of 2.5 g/kg of diet as an indigestible dietary marker to determine the nutrient and energy digestibility in Experiment 1.

**Table 1:** Composition of diets†.

**Ingredient Composition**	**g/kg**
Corn	529.4
Soybean meal (48% CP)	293.8
Dried distillers grains with solubles	120.0
Soybean oil	10.8
DL-methionine	2.2
Bio-Lys^1^	4.4
Salt	4.0
Limestone	13.0
Di-calcium phosphate	12.7
Choline chloride (60%)	1.0
Vitamin and mineral premix^2^	6.3
Titanium dioxide	2.5
**Chemical Composition (calculated)**	**g/kg**
ME (MJ/kg)	12.24
Crude protein	222.5
Ether extract	46.2
Crude fiber	31.1
Calcium	9.0
Non-phytate phosphorus	3.8
Digestible methionine + cysteine	8.5
Digestible lysine	12.0
Digestible threonine	7.5
**Chemical Composition (analyzed)**	**g/kg**
Crude protein	227.4
Ether extract	41.4
Crude fiber	28.3

1 Contained 50.7% of L-lysine in the form of L-lysine sulfate, 0.1% Methionine, 0.1% Cystine, 0.3% Threonine, 0.1% Tryptophan, 0.6% Arginine, 0.3% Isoleucine, 0.5% Leucine, and 0.4% Valine.

2 Provided per kg of diet: Selenium-250 µg; Vitamin A (retinyl palmitate)-4.54 mg; Vitamin D_3_ (cholecalciferol)-0.069 mg; Vitamin E (alpha-tocopherol)-11.94 mg; Menadione- 1.1 mg; Vitamin B_12_-12 µg; Biotin-41 µg; Choline-447 mg; Folic acid-1.4 mg; Niacin-41.3 mg; Pantothenic acid-11 mg; Pyridoxine-1.1 mg; Riboflavin-5.5 mg; Thiamine-1.4 mg; Iron-282 mg; Magnesium-125 mg; Manganese-275 mg; Zinc-275 mg; Copper-27.5 mg; Iodine-844 µg.

† Diets were fed to broiler chickens from 15 to 21 days of age in Experiment 1 and from 1 to 21 days of age in Experiment 2.

### Sample collection

In the first experiment, excreta samples were collected by the batteries for the last 48 hr of the experimental period, by placing clean trays under each cage. All birds were euthanized by carbon dioxide asphyxiation on day 21 and dissected to collect ileal digesta contents. The section of ileum was determined from the point of Meckel's diverticulum to ileo-cecal junction. After exposing the ileum, the digesta samples of chicks in the same battery were squeezed into a sterile plastic bag by applying gentle pressure. The collected ileal digesta and excreta samples were stored at −20°C until further analysis.

In Experiment 2, eight birds were randomly selected per treatment on day 21 and were euthanized by carbon dioxide asphyxiation. Blood samples were collected individually through cardiac puncture and were set at room temperature for 2 hr. The blood samples were then centrifuged at 2,500×g for 30 min at 4°C to separate serum which was stored at −80°C until further analysis. The lumen of ileum was exposed and flushed with ice-cold saline to free it from food particles. The ileal mucosa was scraped using a glass slide, placed into micro-centrifuge tubes and snap frozen using liquid nitrogen. After exposing the distal ends of ceca to the point of attachment at the ileo-cecal junction, cecal contents from both ceca were squeezed into plastic vials and the vials were snap frozen in liquid nitrogen. Ileal mucosal scrapings and cecal contents were then stored at −80°C until further analysis.

### Digestibility assays

Excreta and digesta samples were dried at 65°C for 3 days [15] using a convection oven (Yamato Scientific America Inc., CA) and ground through a 1.0 mm screen (Brinkmann Instruments Inc., NY). The feed samples were oven (Yamato Scientific America Inc., CA) dried at 100°C for 24 hr, and ground through 0.5 mm screen (Brinkmann Instruments Inc., NY). All samples were analyzed in duplicate. Assays for AIDC of AA were carried out on the digesta and diet samples for methionine (Met), cysteine (Cys), lysine (Lys), threonine (Thr), leucine (Leu), isoleucine (Ile), aspartate (Asp), glutamate (Glu), alanine (Ala), glycine (Gly), proline (Pro), and valine (Val). Concentrations of AA in digesta and diet samples were determined after acid hydrolysis in 6 *N* HCl in the presence of phenol at 110°C for 24 hr [16]. Total sulfur AA (TSAA) content was determined after performic acid oxidation followed by acid hydrolysis [16]. Excreta, digesta and diet samples were assayed for nitrogen (N) by micro-Kjeldahl method [17] on a Kjeltech 1028 distilling unit (Foss Inc., MN). From the N concentration of digesta and diet samples, the concentrations of CP were calculated using a multiplication factor of 6.25 [18]. Retention of N was calculated as the difference between N intake and N in the excreta [19]. For digesta and diet samples, the concentration of starch was determined using Total Starch kit (Megazyme International, Ireland) for both digesta and diet samples. Complete solubilization of starch was achieved by cooking samples in the presence of thermo-stable α-amylase followed by amyloglucosidase hydrolysis to glucose, while maltodextrins were hydrolyzed to glucose by glucoamylase. Glucose produced was measured using glucose oxidase/peroxidase reagent [20]. Excreta, digesta and diet samples were assayed for the concentration of TiO_2_ [21], to calculate apparent ileal AA, CP and starch digestibility [22]. Gross energy was determined using an adiabatic oxygen bomb calorimeter (Parr Instrument Co., IL) for diet and excreta samples. The AMEn was calculated by modifying the equation proposed by Scott et al. [23], by replacing chromic oxide marker with TiO_2_ [21], [24].

### Ileal brush border digestive enzyme activity

Total protein was extracted from the ileal mucosal scrapings to measure the activities of maltase (EC 3.2.1.20), sucrase (EC 3.2.1.48) and LAAP (3.4.11.2). Briefly, a buffer mixture, containing phosphate buffer saline (pH 7.2), 0.1% triton, aprotinin (2 µg/ml), leupeptin (2 µg/ml), pepstatin (1 µg/ml), sodium orthovanadate (1 mM), and phenylmethylsulfonyl fluoride (1 mM), was added at 4 mL to 0.5 g of each of the mucosal samples. The buffer added samples were then centrifuged at 10,000 rpm for 10 min at 4°C and the supernatant was transferred to 5 ml tubes. The protein concentration was determined using Pierce BCA Protein assay kit (Thermo Scientific, IL) and expressed in g/dl. The samples were maintained in microcentrifuge tubes at −80°C until they were used for further analysis. The activity of maltase and sucrase were assayed colorimetrically at 540 nm using a spectrophotometer (Biotek Instruments Inc., VT), by measuring μ moles of glucose released per min per g of protein from maltose and sucrose, respectively [25]. The activity of LAAP was determined colorimetrically at 384 nm using a spectrophotometer (Biotek Instruments Inc., VT), by measuring the amount of enzyme activity liberating one μmoles of L-alanine p-nitroanilide per min per g of protein from 4-Nitroaniline [26].

### Cecal short-chain fatty acid concentration

Proportions of the SCFA (acetate, propionate and butyrate), as well as the concentration of total SCFA were determined from the cecal contents. Approximately 0.5 g cecal contents were gently squeezed into a micro-centrifuge tube containing 1 ml of 10% meta-phosphoric acid with 0.4 µl of 4-methyl valeric acid per ml added as an internal standard. The solution was thoroughly mixed using a vortex mixer and centrifuged at 5,700×g for 20 min at 4°C. The SCFA content of the supernatant was measured using a HP Agilent 6890 series gas chromatograph (Agilent Technologies Inc., CA) fitted with a HP 5973 series mass spectrometer (Agilent Technologies Inc., CA). The columns (Agilent Technologies, CA) used were HP-free fatty acid polyester stationary phase capillary columns of polyethylene glycol on Shimalite TPA 60/80, measuring 30 m long with a 0.25 mm internal diameter. The parameters were as follows: 1 µl injection volume, 240°C injector temperature, 12.15 psi pressure, with 1.1 ml per min constant flow and helium carrier. The following running conditions in the oven program were used: 80°C initial temperature hold for 5 min, ramp 10°C per min to 240°C and 12 min hold at 240°C. Total SCFA concentration was expressed as μmol per g of wet cecal content, while the proportions of individual SCFA were expressed in mg per g of total SCFA.

### Statistical analysis

The statistical analysis was carried out as a 2×2 factorial arrangement in a completely randomized design in both experiments. Data were analyzed by MIXED procedure of SAS [27] using battery as the experimental unit in the first experiment, and individual chick as the experimental unit in Experiment 2. Student's t-test was used to separate significant least square means with the probability of type-I error (α) set at 0.05. While the significance was accepted at *P*≤0.05, trends were noted at *P*≤0.10.

## Results

### Performance

Feed intake, body weight gain and mortality data were collected to verify typical growth responses in both the experiments, but were not statistically analyzed. The mean body weight gain (g/chick) from 15–21 days for CON, CON + PP, CON + DFM and CON + PP + DFM treatments were 344, 351, 334 and 342 respectively in Experiment 1. The mean body weight gain (g/chick) from 1–21 days for CON, CON + PP, CON + DFM and CON + PP + DFM treatments were 715, 688, 743 and 701 respectively in Experiment 2. The performance data from both experiments were lower in comparison with typical of Ross 308 broiler chickens over this time period (Aviagen, 2011).

### Digestibility

There were interactions (*P*≤0.01) in AIDC between DFM and PP fed groups in the first experiment for starch, CP, and all AA (Met, Cys, Lys, Thr, Leu, Ile, Asp, Glu, Ala, Gly, Pro, and Val) analyzed. Only the critical AA ((Met, Cys, Lys and Thr) will be discussed here as rest of the AA followed the same trend (Table 2). Regardless of dietary supplementation, AIDC of starch was high, ranging from 97% to 99%. When fed alone or in combination, both PP and DFM increased (*P*≤0.01) AIDC of starch compared to the CON diet. Supplementation of either PP or DFM increased the AIDC of CP, but the magnitude was higher with PP. The AIDC for the combination did not differ significantly from either of the additives when fed alone. Both PP and or DFM increased the AIDC significantly compared to the CON diet for all AA. Although no significant differences were observed in the AIDC of Met between either PP or DFM supplementation, the combination reduced the AIDC which was significantly lower than PP alone. Overall, the AIDC of CP and AA were increased (*P*≤0.01) by an average of 12% by PP, 8% by DFM and 10% by the combination.

**Table 2:** Interactions of exogenous protease and phytase (PP) and direct-fed microbial (DFM) on apparent ileal digestibility coefficient†.

Treatments	Starch	CP^2^	Methionine	Cysteine	Lysine	Threonine
CON^1^	0.968^b^	0.78^c^	0.88^c^	0.68^b^	0.85^b^	0.69^c^
CON + PP	0.984^a^	0.86^a^	0.95^a^	0.80^a^	0.91^a^	0.83^a^
CON + DFM	0.989^a^	0.83^b^	0.94^ab^	0.76^a^	0.89^a^	0.79^ab^
CON + PP + DFM	0.989^a^	0.84^ab^	0.93^b^	0.78^a^	0.90^a^	0.80^ab^
SEM	0.003	0.01	0.01	0.02	0.01	0.02
PP *P-value*	≤0.01	≤0.01	≤0.01	≤0.01	≤0.01	≤0.01
DFM *P-value*	≤0.01	0.17	0.02	0.10	0.11	0.05
PP x DFM *P-value*	≤0.01	≤0.01	≤0.01	≤0.01	≤0.01	≤0.01

a,b Least square means in the same column without a common superscript differ significantly, *P*≤0.05.

n = 8 samples per treatment for interactions.

1 Formulated to meet breeder requirements for 14–21 d old broiler chicks except with a reduction of 0.94 MJ/kg of ME.

2 Crude protein.

† Determined in broiler chickens at 21 days of age in Experiment 1.

Interactions were noted as PP, DFM or the combination increased (*P*≤0.01) N retention compared to the CON diet (Table 3). The magnitude of increase was highest and sub-additive in chicks fed the combination of PP and DFM followed by PP alone and then DFM alone. Diets supplemented with either of the additives resulted in increased (*P*≤0.01) AMEn (Table 3). Only main effects were observed for AMEn as no interaction was observed.

**Table 3:** Effects of exogenous protease and phytase (PP) and direct-fed microbial (DFM) on nitrogen retention (NR) coefficient and nitrogen-corrected apparent metabolizable energy (AMEn)†.

Treatments	NR Coefficient	AMEn
CON^1^	0.64^d^	3143
CON + PP	0.79^b^	3246
CON + DFM	0.75^c^	3225
CON + PP + DFM	0.81^a^	3384
SEM	0.01	32
PP x DFM *P-value*	≤0.01	0.39
No PP	0.69^b^	3184^b^
PP	0.80^a^	3315^a^
SEM	0.01	23
PP *P-value*	≤0.01	≤0.01
No DFM	0.72^b^	3195^b^
DFM	0.78^a^	3305^a^
SEM	0.01	23
DFM *P-value*	≤0.01	≤0.01

a,b Least square means in the same column without a common superscript differ significantly, *P*≤0.05.

n = 8 samples per treatment for interactions and 16 samples per treatment group for main effects.

1 Formulated to meet breeder requirements for 14–21 d old broiler chicks except with a reduction of 0.94 MJ/kg of ME.

† Determined in broiler chickens at 21 days of age in Experiment 1.

### Ileal brush border digestive enzyme activity

Supplementation of PP increased (*P*≤0.05) ileal brush border maltase, sucrase, and LAAP activity in Experiment 2 (Fig. S1). Although DFM did not significantly alter the activity of any of the brush border digestive enzymes, a trend for increased sucrase activity was observed (*P* = 0.07). No interactions between PP and DFM were noted.

### Cecal short-chain fatty acid concentration

Overall interactions were absent among the dietary treatments. Total SCFA concentration did not differ significantly among the dietary treatments (Fig. S2). Total SCFA concentration (μmol per g of wet cecal content) of chickens fed with and without PP were 61.1±2.0 and 57.8±2.0, whereas with and without DFM were 59.8±2.0 and 59.2±2.0, respectively. The proportion of butyrate was increased (*P*≤0.01) with DFM addition, although there were no significant differences observed in the proportions of either acetate or propionate in Experiment 2.

## Discussion

Published data on the AIDC of AA are in agreement with current data on the effects of PP. Cowieson and Ravindran [1] reported a 3% increase in overall AIDC of AA, ranging from 0.44% for Met to 9.1% for Cys, by supplementing an enzyme cocktail containing protease. Increases in the AIDC of starch by 2.0% and CP by 3.6% were also observed by supplementation of the same enzyme cocktail to broiler diets in a different experiment [28]. Increases in the AIDC of dietary starch, CP, and AA can be mediated by more efficient digestion and absorption of these nutrients as exogenous enzymes complement endogenous digestive enzymes [1]. Exogenous proteases may augment endogenous peptidases by increasing protein digestibility and hydrolyzing proteinaceous anti-nutritional factors such as lectins, trypsin inhibitors and antigenic proteins [4], [29]. Hence, increased ileal nutrient digestibility for chickens fed PP may be related to direct effects on the digestion of nutrient substrates as well as reduced endogenous loss, which include pancreatic and brush border digestive enzymes, mucins and sloughed epithelial cells, owing to degradation of anti-nutritional factors in the diet [1].

The gastrointestinal tract swiftly adapts to dietary changes and the activities of endogenous digestive enzymes are modulated in response to physiological needs, primarily with increased luminal sugar and AA content, rather than constantly maintaining high enzyme activity [30]. Therefore, the increased activity of disaccharidases (maltase and sucrase) and LAAP at the ileal mucosa of chickens fed PP might be attributed to increased substrate presence at the apical membrane due to PP enhanced hydrolysis of dietary nutrients. Furthermore, it has been observed that serine protease removes structural proteins in the cell wall of non-starch polysaccharides, allowing the microbes to gain faster access to the substrates [31]. However, supplementation of PP alone did not alter either total cecal SCFA concentration or the proportions of SCFA in Experiment 2. As expected, absence of these carbohydrase-associated effects in this experiment may be due to the enzymatic combination (protease and phytase) used, as the substrate specificity of these enzymes vary greatly in comparison to carbohydrases [32].

In Experiment 1, supplementation of *Bacillus sp*. DFM in corn-SBM-DDGS-based diets increased the AIDC of starch, CP and AA, which resembles published data using a *Lactobacillus*-based DFM [33], [34]. Santoso et al. [10] reported increased nutrient digestion and utilization due to supplementation of *Bacillus sp*. in broiler chicks, which the authors attributed to secretion of protease, amylase and lipase. Furthermore, *Bacillus sp*. DFM have been reported to enhance development of intestinal villi, which is relevant for efficient nutrient digestion and absorption [35]. Present results reinforce the influence exhibited by *Bacillus sp*. DFM in increasing enterocyte activity and nutrient digestibility [36]. The population of beneficial bacteria such as *Lactobacilli spp*. in the gastrointestinal tract has been found to be increased by feeding *Bacillus sp* [37]. The *Lactobacilli spp* in the intestine may secrete and therefore increase the intestinal amylase activity, which promotes increased digestion and absorption of dietary nutrients [38]. The trend in increased sucrase activity by *Bacillus licheniformis* in Experiment 2 was in agreement with Jin et al. [38], who reported that a *Lactobacillus* culture-based DFM increased the activity of brush border carbohydrases in broiler chickens. The current data suggest that supplemental PP and DFM, independent of each other, increased ileal nutrient digestibility, although the underlying mechanisms may be overlapping as the combination did not result in a fully additive response.

Ghazi et al. [4] observed increased N retention in broiler chickens fed protease-treated SBM in agreement with the results noted in Experiment 1. These authors explained that the increased N retention may arise either directly or indirectly from the inactivation of anti-nutritional factors which interfere with the digestive process. Protease supplementation has also been employed to lower dietary protein level without a reduction in broiler performance, resulting in reduced protein waste and N excretion into the environment [6]. Furthermore, supplemental phytase was found to consistently increase N retention in broilers, independent of dietary phytic acid and non-phytate phosphorus levels [8]. Kumprecht and Zobac [39] reported that inclusion of *Bacillus* based DFM in broiler diets increased N retention, in agreement with current results. The sub-additive response by the combination of PP and DFM in increasing N retention suggests complimentary actions of the additives. Overall, increased N retention by PP, DFM or the combination of PP and DFM is in agreement with increased ileal AA and CP digestibility coefficient in the present experiment.

A growing body of research demonstrates that supplementation of protease along with various exogenous enzymes such as amylase and xylanase increases the dietary AMEn for broiler chickens [1], [40], [41], which is in agreement with current data. The primary reasons attributed are increased digestibility of nutrients, degradation anti-nutritional factors and reduced endogenous loss [42], [43]. Published data indicate that the magnitude of increase in AMEn by protease supplementation in corn-SBM-based diets was greater for diets with low ME compared to high ME [29], [41]. The authors speculated this to the low ME: CP ratio in diets with low ME where excess CP will be catabolized [41]. This is in agreement with current results which were obtained by feeding corn-SBM-DDGS-based diets with a reduction of 0.94 MJ/kg of ME. Furthermore, the results of AMEn are in agreement with published data on the effects of exogenous protease in increasing AMEn [28]. However, published data on the direct effects of *Bacillus sp*. DFM on AMEn of broiler chickens are scarce. Increased total SCFA and acetate concentration were observed in broiler chickens fed *Bacillus sp*. DFM for 80 days, while no difference in propionate or butyrate concentrations were observed [11]. Although this is not the same result observed in Experiment 2, a significant effect of the DFM on butyrate proportion was observed. In addition to being used as the major substrate for energy production, butyrate has been observed to be the most effective SCFA to promote the proliferation and functional maturation of intestinal epithelial cells resulting in increased arterial blood flow linked to increased nutrient absorption [44]. Butyrate modifies the motility of upper gastrointestinal wall mediated by polypeptide YY, which induces relaxation of the proximal stomach and lower esophageal sphincter, resulting in decreased transit time and reduced emptying [44]. These physiological functions of butyrate in increasing nutrient digestion and absorption could be responsible for the increased AIDC of nutrients and AMEn by the addition of DFM in Experiment 1.

In conclusion, supplemental PP or *Bacillus*-based DFM increased the AIDC for starch, CP and AA, as well as total N retention through independent but complementary mechanisms in broiler chickens. The effects of PP were also evident on the up-regulation of brush border digestive enzyme activity, possibly due to increased substrate presence at the apical membrane. Supplementation of DFM increased cecal butyrate proportion which could be linked to increased energy utilization and mucosal integrity. The effects of PP and DFM were additive for AMEn and could be mediated at the small intestine for PP and through modifications of hindgut fermentation for DFM.

## Supporting Information

Figure S1 Main effects of exogenous protease and phytase (PP) and direct-fed microbial (DFM) on ileal brush border digestive enzyme activity of broiler chickens at 21 days of age in Experiment 2. Least square mean columns without a common superscript ^a,b^ differ significantly, *P*≤0.05. n = 16 samples per group for main effects.
(TIF)

Figure S2 Main effects of exogenous protease and phytase (PP) and direct-fed microbial (DFM) on cecal short-chain volatile fatty acid proportion in broiler chickens at 21 days of age in Experiment 2. Least square mean columns without a common superscript ^a,b^ differ significantly, *P*≤0.05. n = 16 samples per group for main effects.
(TIF)

Data S1 Original replicate data for Tables 2 and 3 from Experiment 1 and Figures S1 and S2 from Experiment 2. CON  =  Control; CON + PP  =  Control with Protease and Phytase enzymes; CON + DFM  =  Control with Direct-fed microbial; and CON + PP + DFM  =  Control with Protease, Phytase and Direct-fed microbial. n = 8 data per treatment.
(XLSX)
