# Supplementary figures and images for: Effects of Protease, Phytase and a Bacillus sp. Direct-Fed Microbial on Nutrient and Energy Digestibility, Ileal Brush Border Digestive Enzyme Activity and Cecal Short-Chain Fatty Acid Concentration in Broiler Chickens
Source: PLoS One. 2014 Jul 11;9(7):e101888. doi: 10.1371/journal.pone.0101888 (PMC4094469; doi:10.1371/journal.pone.0101888)

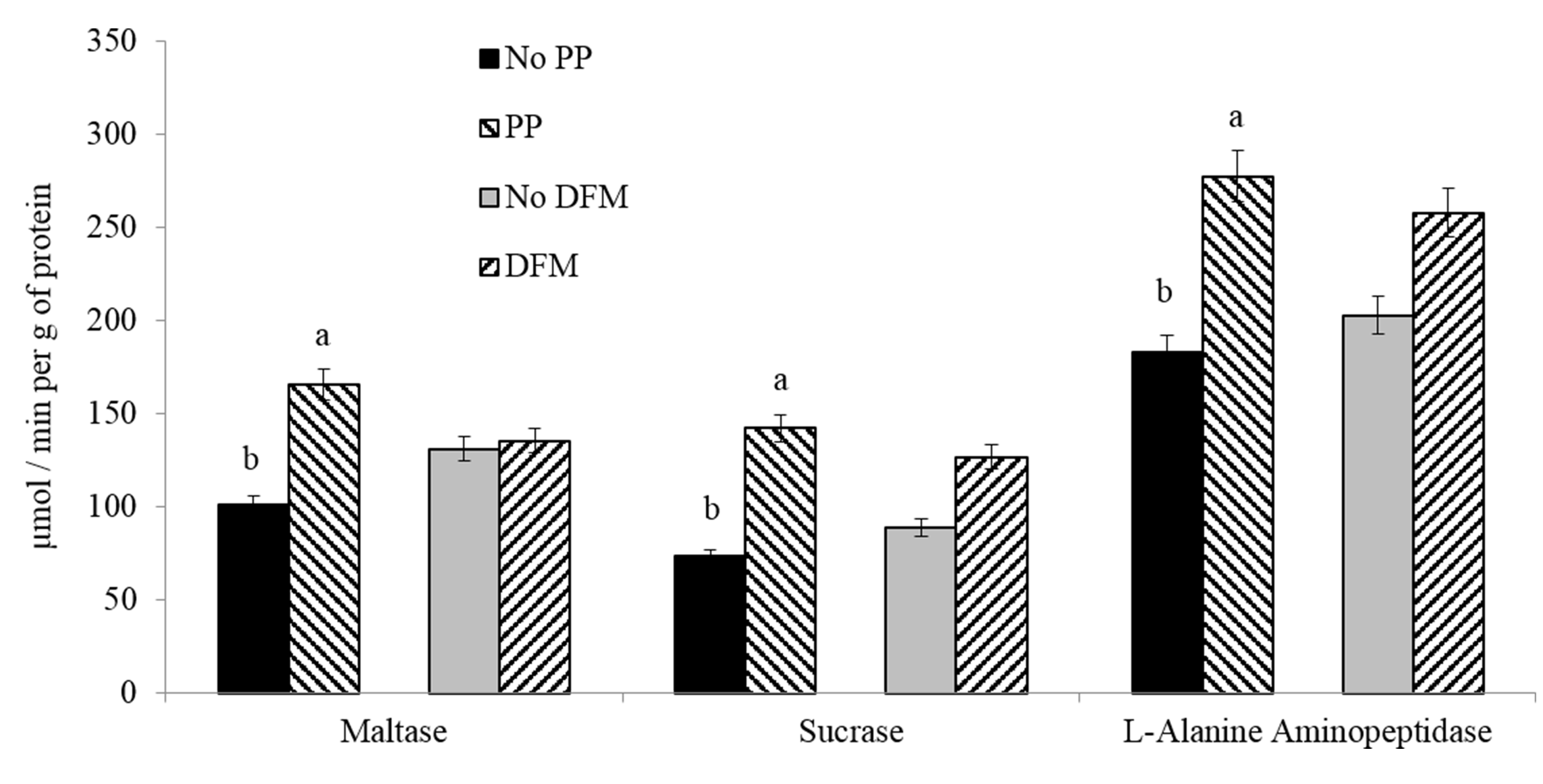

Supplement: Figure S1 — Main effects of exogenous protease and phytase (PP) and direct-fed microbial (DFM) on ileal brush border digestive enzyme activity of broiler chickens at 21 days of age in Experiment 2. Least square mean columns without a common superscript a,b differ significantly, P≤0.05. n = 16 samples per group for main effects. (TIF) [file pone.0101888.s001.tif]

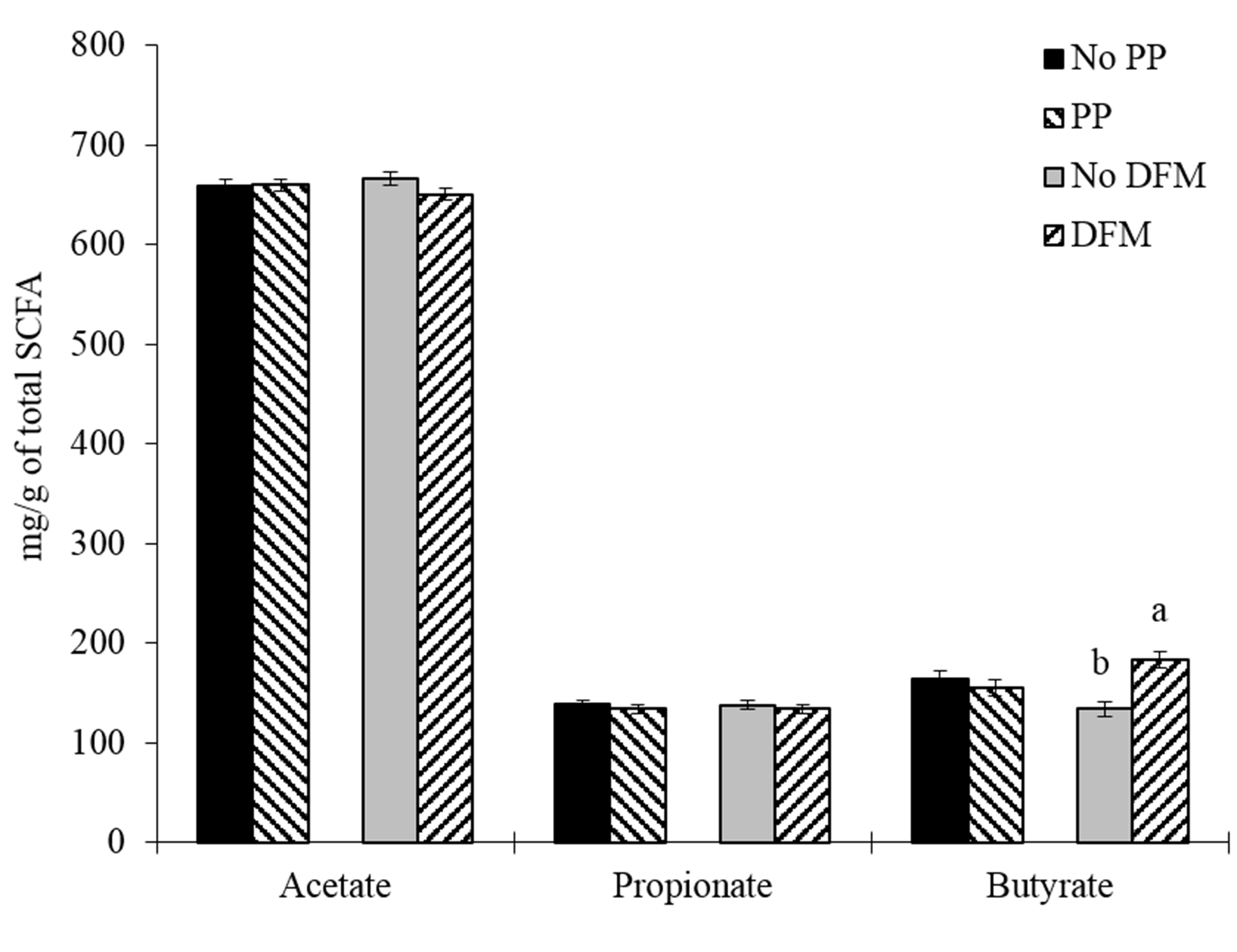

Supplement: Figure S2 — Main effects of exogenous protease and phytase (PP) and direct-fed microbial (DFM) on cecal short-chain volatile fatty acid proportion in broiler chickens at 21 days of age in Experiment 2. Least square mean columns without a common superscript a,b differ significantly, P≤0.05. n = 16 samples per group for main effects. (TIF) [file pone.0101888.s002.tif]
